# The Role of Sulphonic and Phosphoric Pendant Groups on the Diffusion of Monovalent Ions in Polyelectrolyte Membranes: A Molecular Dynamics Study

**DOI:** 10.3390/membranes11120940

**Published:** 2021-11-28

**Authors:** Ismail Abdulazeez, Billel Salhi, Nadeem Baig, Qing Peng

**Affiliations:** 1 Interdisciplinary Research Center for Membranes and Water Security, King Fahd University of Petroleum and Minerals, Dhahran 31261, Saudi Arabia; billel@kfupm.edu.sa (B.S.); nadeembaig@kfupm.edu.sa (N.B.); 2 Physics Department, King Fahd University of Petroleum and Minerals, Dhahran 31261, Saudi Arabia; 3 KACARE Energy Research and Innovation Center at Dhahran, Dhahran 31261, Saudi Arabia; 4 Hydrogen and Energy Storage Center, King Fahd University of Petroleum and Minerals, Dhahran 31261, Saudi Arabia

**Keywords:** lithium-ion recovery, brine, polyelectrolyte membrane, electrodialysis, MD simulation

## Abstract

Lithium-ion consumption has risen significantly in recent years due to its use in portable devices. Alternative sources of lithium, which include the recovery from brine using the sustainable and eco-friendly electrodialysis technology, has been explored. This technology, however, requires effective cation-exchange membranes that allow the selective permeation of lithium ions. In this study, we have investigated, via molecular dynamics simulations, the role of the two common charged groups, the sulfonic and the phosphoric groups, in promoting the adsorption of monovalent ions from brine comprising Li^+^, Na^+^, Mg^2+^, and Ca^2+^ ions. The analysis of the mean square displacement of the ions revealed that Li^+^ and Na^+^ ions exhibit superior diffusion behaviors within the polyelectrolyte system. The O-atoms of the charged groups bind strongly with the divalent ions (Mg^2+^ and Ca^2+^), which raises their diffusion energy barrier and consequently lowers their rate of permeation. In contrast, the monovalent ions exhibit weaker interactions, with Na^+^ being slightly above Li^+^, enabling the permeation of Li^+^ ions. The present study demonstrates the role of both charged groups in cation-exchange membranes in promoting the diffusion of Li^+^ and Na^+^ ions, and could serve as a guide for the design of effective membranes for the recovery of these ions from brine.

## 1. Introduction

The advent of green energy-storage devices, such as lithium-ion batteries (LIBs), which are gradually phasing out the existing lead-acid batteries, nickel-cadmium batteries, and nickel-metal hydride batteries, has contributed significantly to the technological advancement of the world [1,2,3]. LIBs have found applications in several portable electronic devices, such as cell phones, camcorders, laptops, and, more recently, in electric cars [4,5]. The traditional method of production of lithium-ions is through mining or the acid leaching of lithium aluminum inosilicates, otherwise known as spodumene, LiAl(SiO_3_)_2_ [6]. Lithium metals with high purity (99.50–99.99%) have also been produced at high temperatures by the electrowinning of molten eutectic LiCl-KCl salts [7,8]. These technologies are, however, energy-intensive, capital-intensive, and often result in the production of non-ecofriendly side products. Sodium-ion batteries (SIBs) have also emerged as promising substitutes for LIBs, due to the similarity in their valence shell chemistry [9,10,11]. In addition, sodium is cheap, non-toxic, and relatively abundant, compared to lithium. While research attention is gradually shifting to the development of SIB technology, it is critical to continue to find alternative sources of the metal ions for the sustainability of these technologies.

Electrodialysis (ED) is a membrane-desalination technology that utilizes ion-exchange membranes to transport ions under the influence of an applied electric potential [12,13,14]. While this technology is used mainly for water purification, it has the potential for the recovery of lithium ions from brine, especially since most brine contains, in addition to Na^+^ ions, an abundance of cations, such as Ca^2+^, Mg^2+^, K^+^, and L^i+^ ions [15,16,17,18]. The core of this technology is, thus, the design of effective monovalent cation-exchange membranes that allow the selective permeation of Li+ ions while rejecting the divalent ions. Typical cation exchange membranes are composed of polyelectrolyte (PE) molecules containing sulfonic acid, phosphoric acid, or carboxylic acid groups [19]. The interaction of the active functional groups and the mobility of the molecular chain of the PE play critical roles in the diffusion of the ions through the membrane. There is, therefore, the need for a careful architecture of the polymeric backbone and the active functional groups, as these affect the overall performance of the cation-exchange membranes.

Molecular simulation is a functional tool in the design of effective polyelectrolytes for the selective permeation of various metal ions. It is reported in various literature as being complementary to the experimental approach, and has the advantages of low-cost and less labor, and yields results with high accuracy. Several reports have documented the use of simulations to investigate the mechanisms of interactions and the diffusion of counterions in polymeric materials [20,21,22,23,24]. The polyelectrolyte–water molecular simulation model proposed by Stevens and Kremer [25,26] revealed that the spatial orientation of PE molecules in aqueous media was non-linear; rather, they appeared coiled, and shrunk with the aggregation of counterions. The nature of the aggregation of the counterions and the effect of the molecular chain length was later reported by Liu et al. [27]. The nature of aggregation of the counterions was also studied by Konieczny et al. [28], and they found that a few counterions were confined around the PE molecules, while others were distributed in the aqueous phase. The role of co-existing counterions, Ca^2+^ ions, on the distribution of Na^+^ ions around the PE molecules in the aqueous phase was investigated by Molnar et al. [29], and they found that the Ca^2+^ ions shielded the intrinsic negative charges on the PE molecules, preventing the effective distribution of Na^+^ ions and promoting the shrinkage of the molecules. The interaction of co-existing Na^+^ and Ca^2+^ ions on polymethacrylic acid-based PE molecules was reported by Huang et al. [30,31]. Their results revealed that the attraction of the carboxylic acid groups towards Ca^2+^ ions on the PE molecules played a significant role in the geometric size of the PE molecules. Sun et al. [32] recently proposed the PE chain–counterion aqueous solution model for the investigation of the diffusion behavior of Li^+^ and Mg^2+^ ions on sulfonic and phosphoric acid fixed-charged group PEs. Their results revealed that the interaction of the counterions with the fixed-charged groups determined the diffusion of the ions, and that the sulfonic acid group was ideal for the selective permeation of monovalent ions, such as Li^+^ ions. This model, owing to its remarkable consonance with experimental data as reported by Sun et al. [32], is further explored in this study.

In the present study, we have investigated, using molecular dynamics (MD), simulations of the influence of both the sulfonic and the phosphoric acid fixed-charge groups on the diffusion of Li^+^, Na^+^, Ca^2+^, and Mg^2+^ ions on PE membranes in aqueous media. The nature of the interactions and the mechanism of diffusion of the counterions through the membrane material were fully accounted for. This study revealed the role of both functional groups in promoting the adsorption of monovalent ions, and could serve as a basis for the design of effective ion-exchange membranes for the recovery of Li^+^ and Na^+^ ions from brine.

## 2. Molecular Dynamics Simulations

### 2.1. Model

**Figure 1:**
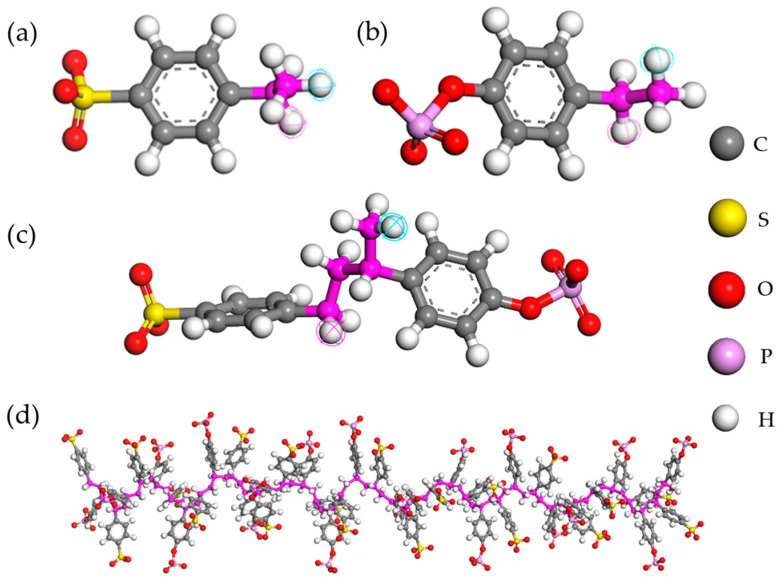
Structural conformations of (**a**) 4-ethyl benzene sulfonate (M_1_), (**b**) 4-ethyl-phenyl phosphate (M_2_), (**c**) 4-[3-(4-phosphonoxy-phenyl)-butyl]-benzene sulfonic acid (co-M_1_M_2_), and (**d**) the polyelectrolyte chain showing the pendant groups.

The structural backbone of typical cation-exchange membranes generally contains fixed-charge acid groups, mainly the sulfonic, the phosphoric, or the carboxylic acid groups. The present membrane material was constructed by mixing two aromatic moieties containing the sulfonic and the phosphoric groups. Initially, the two monomers, namely, 4-ethylbenzene sulfonate (designated as M_1_) and 4-ethyl-phenyl phosphate (designated as M_2_), as presented in Figure 1a,b, were sketched and geometrically optimized. The two monomers were co-polymerized to form the copolymer 4-[3-(4-phosphonoxy-phenyl)-butyl]-benzene sulfonic acid (designated as co-M_1_M_2_), with the degree of polymerization of 1, as presented in Figure 1c, using the Block co-polymer command. Thereafter, co-M_1_M_2_ was taken as the monomer for the building of the homopolymer –[M_1_M_2_]–_20_, with the degree of polymerization of 20, as presented in Figure 1d. All the molecules—the monomers, the copolymer, and the final polyelectrolyte homopolymer—were geometrically optimized using the Condensed-phase Optimized Molecular Potentials for Atomistic Simulation Studies II (COMPASS II) forcefield on the Forcite module. The COMPASS II forcefield was selected because it provided extended coverage of the COMPASS forcefield to polymeric and heterocyclic systems [33]. The metal cations Li^+^, Na^+^, Ca^2+^, and Mg^2+^, and the anion Cl^−^ ions, were built and the respective charges were assigned. The forcefields assigned for the ions were lithium, +1 ion for Li^+^; sodium, +1 ion for Na^+^; calcium, +2 ion for Ca^2+^; magnesium, +2 ion for Mg^2+^; and chlorine, −1 ion for Cl^−^. The water molecules were modeled and a rigid planar simple-point charge SPC-like water model was obtained [34,35]. The forcefield selected for the atoms were h1o, hydrogen-bonded to O, F, and o2*, oxygen, sp3 in water explicitly for the hydrogen and oxygen atoms, respectively.

### 2.2. Construction of Polyelectrolyte–Aqueous System

**Figure 2:**
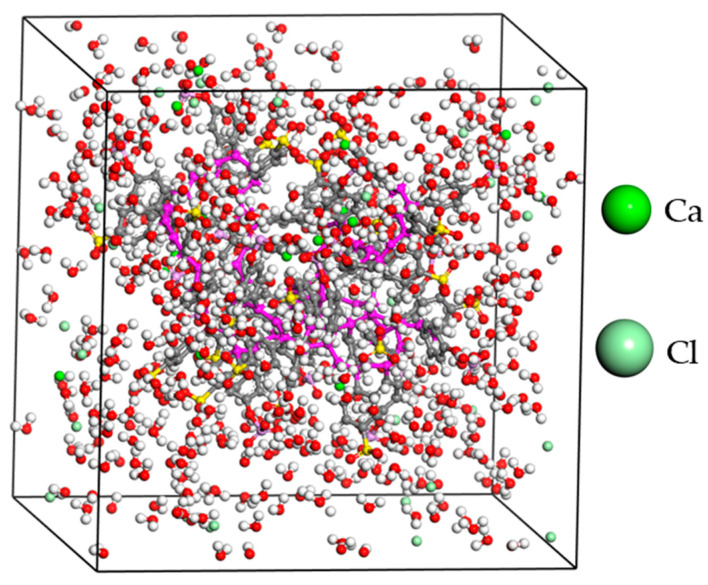
Model of polyelectrolyte-CaCl_2_-water molecules at 298 K. The purple region represents the polyelectrolyte backbone. All other atoms remain the same as depicted in Figure 1.

The simulation box was built using the amorphous cell module. The polyelectrolyte-metal chloride aqueous system was modeled as amorphous in a cubic cell with an edge length of 30 Å. The periodic boundary conditions are applied in the three orthogonal directions. The density of the system was set to 1.05 g/cm^3^, the temperature to 298 K, and the convergence tolerance quality was set as ultrafine. A typical simulation box consisted of the polyelectrolyte chain with a degree of polymerization 20, 20 cations (Li^+^, Na^+^, Ca^2+^ or Mg^2+^), a balanced charge of chloride ions (1:1 ratio for the monovalent ions and 1:2 for the divalent ions), and 500 molecules of water. The constructed simulation box was geometrically optimized using the Forcite module and the system maintained electrical neutrality, as presented in Figure 2 for the PE-CaCl_2_-H_2_O system.

### 2.3. Molecular Dynamics Simulations

**Figure 3:**
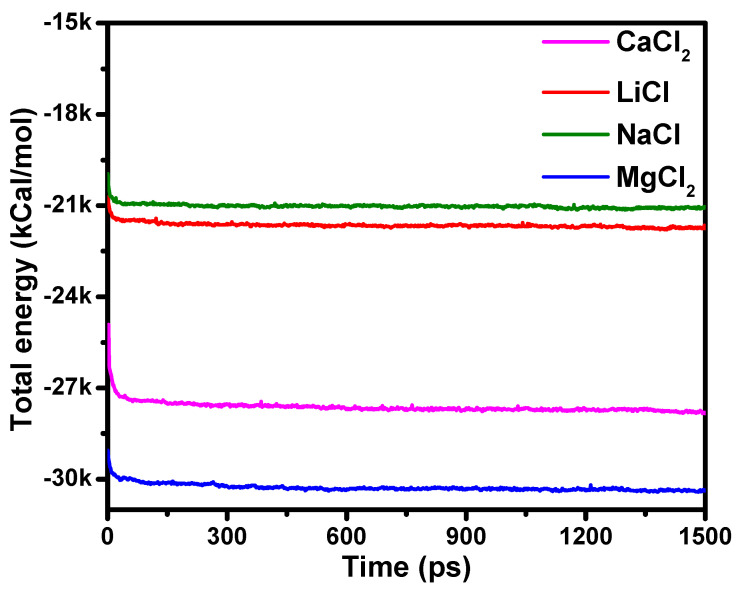
Total energy variations within the NVT ensemble for the polyelectrolyte chain–aqueous system comprising CaCl_2_, LiCl, NaCl, and MgCl_2_ salts.

Molecular dynamics simulations were conducted using the Forcite module. The initial systems were structurally optimized using the congruent gradient, followed by a dynamics simulation on the constant pressure-constant temperature (NPT) ensemble (P = 10^−4^ GPa, T = 298 K) to stabilize the system with a simulation time of 500 ps and a time step of 0.001 ps. The molecules of the polyelectrolyte chain from the last frame generated from the NPT simulation were fixed using the constraints toolbar, and the cations were indicated as set. The system was subjected to the dynamics simulation under the constant volume-constant temperature (NVT) ensemble with a total simulation time of 1.5 ns, and the trace file recording was output at every 5000 steps (5 ps). The starting velocity of the atoms at a given temperature was designated randomly by the Boltzmann distribution, the summation method was atom-based, and a truncation radius of 18.5 Å was chosen for the non-bonded interaction energy. The Particle–Particle Particle–Mesh (PPPM) method of summing up the coulombic electrostatic potential energy was chosen, and the Berendsen constant temperature thermal bath and the constant pressure system was selected for temperature and pressure control. The total energy of the system under the NVT ensemble was determined to confirm the attainment of equilibrium. The total energies of the CaCl_2_, LiCl, NaCl, and the MgCl_2_ systems fluctuated around 27,000, 21,700, 21,000, and 30,000 kCal/mol, respectively, as presented in Figure 3.

The diffusion properties of the ions within the polyelectrolytes were determined by estimating the mean square displacement (MSD). The MSD indicates the average separation of all particles from their corresponding initial positions at time *t* into the motion. The greater the MSD value, the higher the diffusivity of the ions within the system. The MSD at time *t* in a given ensemble is expressed by the Equation:

(1) $$\mathrm{MSD}=\frac{1}{N}\sum_{i=1}^{N}{\left|x_{i}\left(t\right)-x_{i}\left(0\right)\right|}^{2}$$

where *N* represents the number of particles to be averaged, and *x_i_*(0) and *x_i_*(*t*) are the initial positions of the *i* ion and the position of the ion at time *t*, respectively.

The diffusion coefficient of the ions, *D*, was estimated following the Einstein Equation, as follows:

(2) $$D=\frac{1}{6}\;\underset{t\to \infty }{\lim}\frac{d\left(\mathrm{MSD}\right)}{dt}$$

A plot of log (MSD) vs. log *t* should give a slope of unity, which, when divided by six, yields the diffusion coefficient of the ions.

The description of the counterion distribution in the vicinity of the fixed-charge groups was estimated by the pair correlation function (PCF), using the expression:

(3) $$g_{xy}\left(r\right)=\frac{V\left[\sum_{i\neq j}\delta \left(r-\left|r_{xi}-r_{yj}\right|\right)\right]}{\left(N_{x}N_{y}-N_{xy}\right)4\pi r^{2}dr}$$

where *x* and *y* depict the two interacting systems, *V* represents the volume of the system, *r* the separation between them, *N_x_* and *N_y_* the number of particles of *x* and *y*, and where *N_xy_* stands for the number of similar *x* and *y* particles and *r_xi_* and *r_yj_* for the 3D coordinates of *x* in *i* and *y* in *j*, respectively.

The interaction energies of the counterions towards the fixed-charge groups were estimated as follows:

(4) $$\Delta E_{int}=E_{xy}-\left(E_{x}+E_{y}\right)$$

where *E_xy_* represents the total energy of the entire system, and *E_x_* and *E_y_* are the energies of the PE–aqueous system and the counterions, respectively.

## 3. Results and Discussion

### 3.1. The Role of the Fixed-Charged Groups

**Figure 4:**
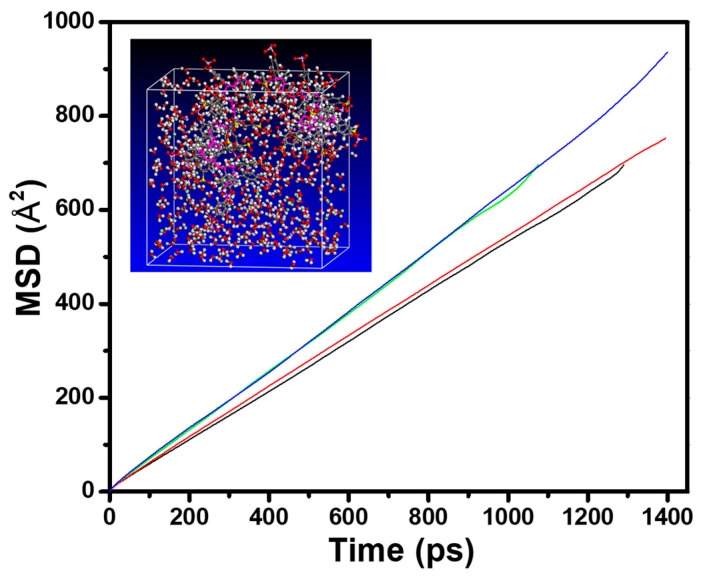
Mean square displacement as a function of time (MSD–*t* curves) of Li^+^ (**blue**), Na^+^ (**green**), Mg^2+^ (**red**), and Ca^2+^ (**black**) ions in aqueous polyelectrolyte systems. All the atoms remain the same as depicted in Figure 1.

A MD simulation was conducted to investigate the counterion migration within the polyelectrolyte membrane material. A system comprising the polyelectrolyte, 500 molecules of water, 20 cations, and charge-balanced Cl^−^ ions were constructed and subjected to NVT dynamic simulation. The diffusion of the ions through the membrane were analyzed by the mean square displacement (MSD) analysis. The MSD–*t* curves of the polyelectrolyte–aqueous system comprising Li^+^, Na^+^, Mg^2^+, and Ca^2^+ ions are presented in Figure 4. The results revealed that the MSD of the monovalent ions (Li^+^ and Na^+^) increases linearly with time, implying the diffusion of these ions within the polyelectrolyte system. The divalent ions (Mg^2+^ and Ca^2+^), in contrast, exhibited a relatively stronger interaction with the charged groups on the polyelectrolyte promoted by their effective nuclear charges. Compared to the previous report on isolated sulfonic and phosphoric fixed-charge systems [32], the present polyelectrolyte–aqueous system comprising both functional groups on the polyelectrolyte chain exhibited a weaker attraction for the monovalent ions, resulting in higher MSD values. This could serve as a strategy for the selective permeation of the monovalent ions during recovery from a mixture of all the ions, such as in brine.

### 3.2. Diffusion of Counterions in Aqueous Media

**Figure 5:**
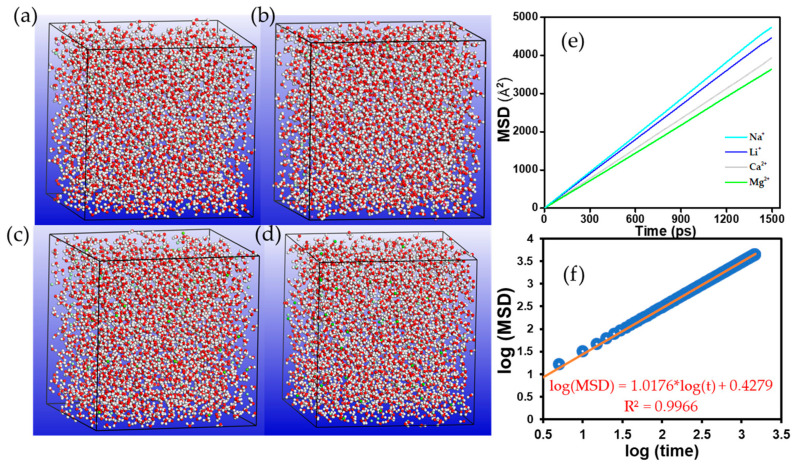
Representative simulation model of the counterions–aqueous system (**a**) Li^+^, (**b**) Na^+^, (**c**) Mg^2+^, and (**d**) Ca^2+^ ions at 298 K. The MSD–*t* curve of all the ions—blue (Li^+^), red (Na^+^), black (Mg^2+^), and green (Ca^2+^)—are shown in (**e**), whereas the log (MSD) vs. log (t) curve for Li^+^ ions is shown in (**f**). All other atoms remain the same as depicted in Figure 1.

The role of the polyelectrolyte chain on the diffusion of the metal ions was further investigated by carrying out MD simulations in an aqueous system without the polyelectrolyte. The simulation box in Figure 5a–d consisted of 2500 molecules of H_2_O, 50 cations, and a balanced charge of Cl^−^ ions to maintain electrical neutrality. The density of the aqueous system was set to 1.05 g/cm^3^ while the other simulation conditions were maintained as before. The mean square displacement as a function of time (MSD–*t*) curves of the NVT simulation (Figure 5e) revealed that the MSD of all the cations increased linearly with time and followed the order Na^+^ > Li^+^ > Ca^2+^ > Mg^2+^, in accordance with their hydration radii [36]. The log (MSD) vs. log (*t*) curve for the Li^+^ ions (Figure 5f) and for the other ions (Supplementary Materials) yielded linear fits in all cases, with slopes of 1.0176, 1.0216, 1.0140, and 1.0154 (almost unity) for Li^+^, Na^+^, Mg^2+^, and Ca^2+^ ions, respectively, suggesting that the migration of the ions in aqueous solution occurs predominantly through the diffusion process. The corresponding diffusion coefficients, D, of the ions were calculated as 1.696 × 10^−9^, 1.702 × 10^−9^, 1.690 × 10^−9^, and 1.692 × 10^−9^ m^2^/s for Li^+^, Na^+^, Mg^2+^, and Ca^2+^ ions, respectively. While the simulated D values did not yield exactly the same results as the actual D values of the ions in aqueous media [37], the simulated values are of almost the same magnitude as the actual values, and are consistent with the size–charge correlation, in the order Mg^2+^ < Ca^2+^ < Li^+^ < Na^+^.

### 3.3. Interaction of Co-Existing Ions with the Polyelectrolyte Chain

**Figure 6:**
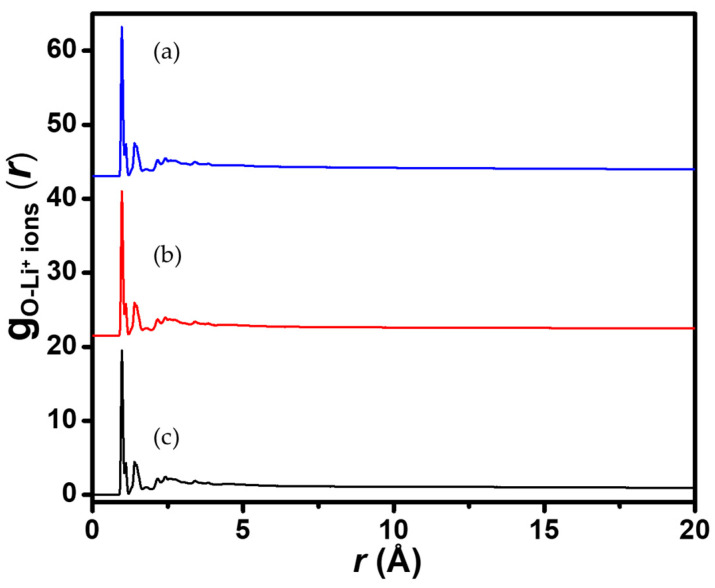
Radial distribution functions of O-atoms in the sulfonic and phosphoric fixed-charge groups to Li^+^ ions in an ensemble comprising (**a**) Li^+^/Na^+^, (**b**) Li^+^/Mg^2+^, and (**c**) Li^+^/Ca^2+^ ions.

**Figure 7:**
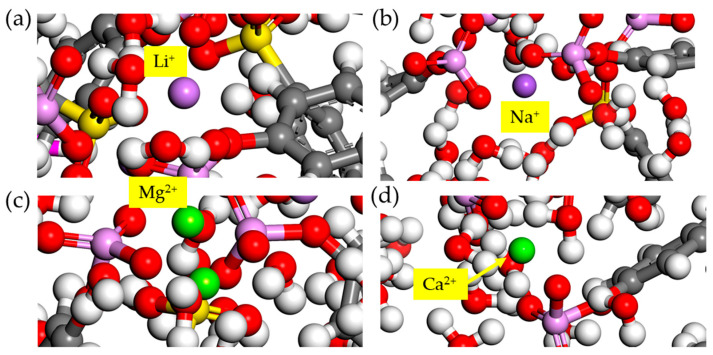
Snapshots of the interactions of (**a**) Li^+^, (**b**) Na^+^, (**c**) Mg^2+^, and (**d**) Ca^2+^ ions during an NVT simulation at 298 K. All other atoms remain the same as depicted in Figure 1.

The interaction of the polyelectrolyte chain with co-existing ionic systems Li^+^/Na^+^, Li^+^/Mg^2+^, and Li^+^/Ca^2+^, was further investigated. MD simulations were carried out in an NVT ensemble on a simulation box consisting of a 1:1 ratio for the ions, the polyelectrolyte chain, 500 molecules of H_2_O, and a balance of Cl^−^ ions. The simulation conditions remained the same as previously. Figure 6 presents the distribution of Li^+^ ions around the charged groups. Two major peaks were observed in all plots, indicating two major interaction regions in accordance with the ionic hydration shells [38,39]. The sharp peak around 1.05 Å corresponds to a strong electrostatic attraction between the O-atoms of the charged groups and the Li^+^ ions. The second peak at 1.65 Å indicates that two major close-range interactions occur between the O-atoms and the Li^+^ ions. For other peaks at higher separations, *r* appeared weaker and eventually diminished due to the shielding of the negative charges, possibly by molecules of water, thereby weakening the interactions with Li^+^ ions. The snapshots of the attraction of the counterions towards the O-atoms are presented in Figure 7, and indicate a moderate attraction for the Li^+^ ions, resulting in the effective diffusion of the ions within the membrane material.

### 3.4. Interaction of the Polyelectrolyte Chain with Mg^2+^/Li^+^ Ions

**Figure 8:**
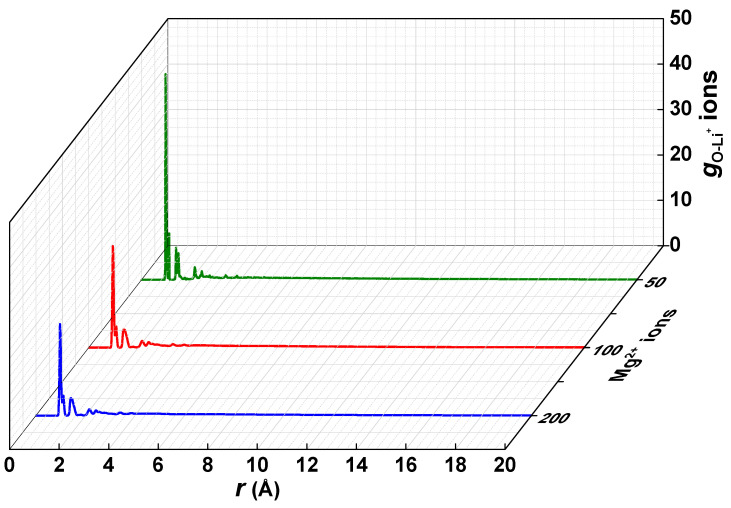
Radial distribution functions of O-atoms in the sulfonic and phosphoric fixed-charge groups to Li^+^ ions in an ensemble comprised of Mg^2+^/Li^+^ ions.

The concentration of Mg^2+^ ions in most brine is often greater than those of Li^+^ ions [40,41,42]. This results in a competition in the diffusion of the ions during the recovery process. In order to suppress the interference of the Mg^2+^ ions, it is desired that the polyelectrolyte chain in the membrane material exhibits a high affinity and, consequently, a greater retardation for the competing ions. The interaction of the polyelectrolyte chain with Mg^2+^/Li^+^ ions was investigated in this study to explore the effect of a high concentration of Mg^2+^ ions on the diffusion of Li^+^ ions. Three PE–aqueous systems were constructed, comprising 5 polyelectrolyte chains, 2500 molecules of H_2_O, 20 Li^+^ ions, 50, 100, and 200 Mg^2+^ ions in the first, second, and third systems, respectively, and a balanced charge of Cl^−^ ions. All other simulation details remained as previously described. The distribution of Li^+^ ions around the charged groups are reflected in Figure 8. Two distinct strong peaks are visible in the curve, similar to those presented in Figure 6. The intensity of the peak at 1.05 Å was strongest when the amount of Mg^2+^ ions was maintained at 50. This is due to the presence of sufficient charged groups, and the competitive adsorption between the Mg^2+^ and the Li^+^ ions was not substantial. Increasing the Mg^2+^ ions to 100, and later to 200, resulted in a decrease in the intensity of the O-Li distribution peak, which occurred as a result of the significant shielding of a considerable amount of Li^+^ ions which are prevented from reaching the electro-adsorption layer of the charged groups. This weakens the interaction with the Li^+^ ions, and promotes their diffusion through the polyelectrolyte. These results suggest that increasing the concentration of the Mg^2+^ ions enhances the diffusion of Li^+^ ions, making the membrane material suitable for the recovery of Li^+^ ions in brine containing high concentrations of Mg^2+^ ions.

**Figure 9:**
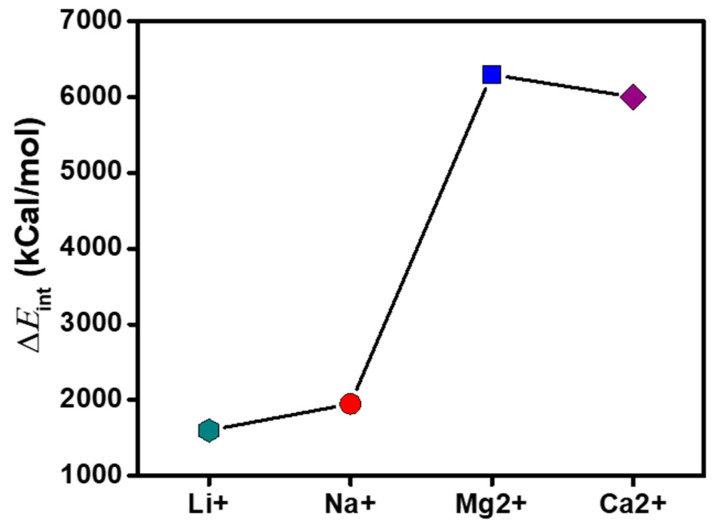
Interaction energy of the counterions towards the sulfonic and phosphoric pendant groups in the polyelectrolyte membrane.

The permeability of the ions was further investigated by constructing an ensemble consisting of the polyelectrolyte chain, 500 molecules of H_2_O, 20 ions each of Li^+^, Na^+^, Mg^2+^, and Ca^2+^, and a balanced charge of Cl^−^ ions. At the end of the NVT dynamic simulation, the final frame was taken and the spatial position of constraints of the polyelectrolyte chain was removed. The total energy of the system, the energies of the solvated counterions, and the energy of the aqueous polyelectrolyte system were determined following a previously reported method [32]. The interaction energies were calculated using Equation (4) and presented in Figure 9. The polyelectrolyte exhibited a significant attraction for divalent ions (Mg^2+^ and Ca^2+^), which increased the energy barrier between them, resulting in the lowering of their diffusion characteristics. The order of interaction energies (Mg^2+^ > Ca^2+^ > Na^+^ > Li^+^) indicates the selective permeation of monovalent ions through the system.

## 4. Conclusions

We have investigated the diffusion of Li^+^, Na^+^, Mg^2+^, and Ca^2+^ ions in brine through the polyelectrolyte membrane materials containing sulfonic and phosphoric pendant groups by means of molecular dynamics simulations. It was revealed that the O-atoms of the charged groups exhibit stronger attractions for the divalent ions, resulting in a rise in the diffusion energy barrier and, consequently, lowering their diffusion through the membrane material. The analysis of the mean square displacement of the ions revealed that Li^+^ and Na^+^ ions exhibit greater values due to the weak attraction by the charged groups. In the presence of higher concentrations of Mg^2+^ ions, the radial distribution function of the O-Li atoms decreases, suggesting the diffusion of Li+ ions through the polyelectrolyte system. This study demonstrates the role of both the sulfonic and the phosphoric pendant groups in promoting the diffusion of monovalent ions. Our results could serve as a guide for the design of effective cation-exchange membranes for the recovery of Li^+^ and Na^+^ ions from brine.

## Data Availability

The raw data generated during this study will be made available by the corresponding authors, without undue reservation, upon request.

## Supplementary Materials

The following are available online at https://www.mdpi.com/article/10.3390/membranes11120940/s1, Figure S1: log (MSD) vs log t plots of (a) Mg^2+^, (b) Ca^2+^ and (c) Na^+^ ions.
