# Supplementary material for: The Role of Sulphonic and Phosphoric Pendant Groups on the Diffusion of Monovalent Ions in Polyelectrolyte Membranes: A Molecular Dynamics Study
Source: Membranes (Basel). 2021 Nov 28;11(12):940. doi: 10.3390/membranes11120940 (PMC8703909; doi:10.3390/membranes11120940)
Supplement: Supplementary file 1 [file membranes-11-00940-s001.zip › membranes-1474718-supplementary.pdf]

The Role of Sulphonic and Phosphoric Pendant Groups on the Diffusion of Monovalent ions in Polyelectrolyte Membranes: A molecular dynamics study

Ismail Abdulazeez [1,*], Billel Salhi [1], Nadeem Baig [1], Isam H. Aljundi [1,2,*] and Qing Peng [3,4,5,*]

[1] Interdisciplinary Research Center for Membranes and Water Security, King Fahd University of Petroleum and Minerals, Dhahran 31261 Saudi Arabia
[2] Chemical Engineering Department, King Fahd University of Petroleum and Minerals, Dhahran 31261 Saudi Arabia
[3] Physics Department, King Fahd University of Petroleum and Minerals, Dhahran 31261 Saudi Arabia
[4] KACARE Energy Research and Innovation Center at Dhahran, Dhahran 31261 Saudi Arabia
[5] Hydrogen and Energy Storage Center, King Fahd University of Petroleum and Minerals, Dhahran 31261, Saudi Arabia

* Correspondence: ismail.abdulazeez@kfupm.edu.sa (IA); aljundi@kfupm.edu.sa (I.H.A); qing.peng@kfupm.edu.sa (QP)

Supplementary Information

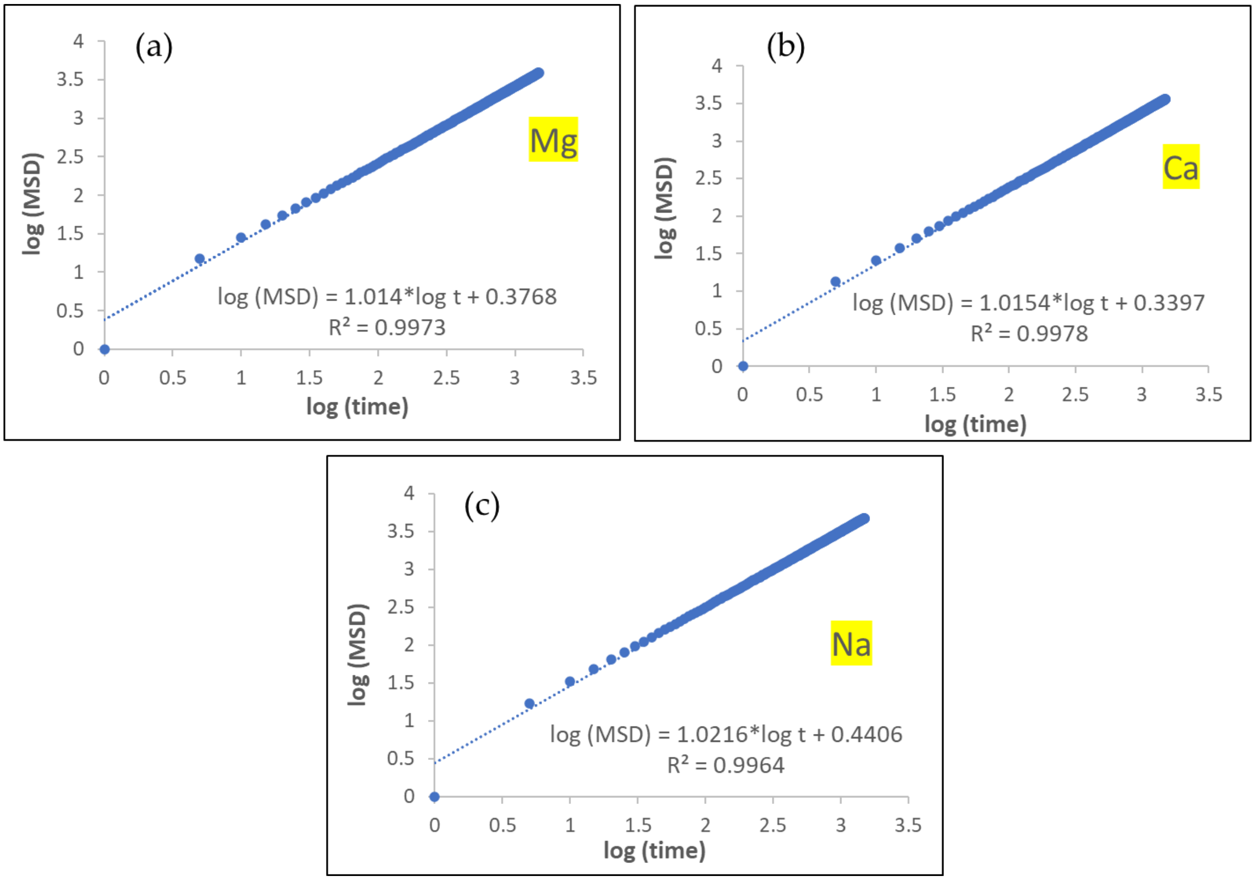

Figure S1. log (MSD) vs log t plots of (**a**) Mg^{2+}, (**b**) Ca^{2+} and (**c**) Na^{+} ions.
